# Antiphospholipid antibody levels in early systemic lupus erythematosus: are they associated with subsequent mortality and vascular events?

**DOI:** 10.1093/rheumatology/kez239

**Published:** 2019-06-30

**Authors:** Charis Pericleous, Amrita D’Souza, Thomas McDonnell, Vera M Ripoll, Oliver Leach, David Isenberg, Ian Giles, Anisur Rahman

**Affiliations:** Division of Medicine, Centre for Rheumatology Research, University College London, London, UK

**Keywords:** systemic lupus erythematosus, anti-phospholipid antibodies, mortality, vascular thrombosis, beta-2-glycoprotein I, anti-phospholipid syndrome, anti-DI

## Abstract

**Objectives:**

aPL are present in between 20 and 30% of patients with SLE. They can cause vascular events (VE) or pregnancy morbidity. aCL and anti-beta-2-glycoprotein I (anti-β2GPI) are measured in clinical practice. Domain I (DI) of β2GPI is the main site for aPL binding. We investigated the prevalence of IgG anti-DI, aCL and anti-β2GPI antibodies in early SLE and their association with mortality and development of VE.

**Methods:**

Samples from 501 patients with SLE that had been obtained and stored early during their disease were tested for IgG anti-DI, aCL and anti-β2GPI antibodies by ELISA. LA status and history of VE were obtained by reviewing medical records. Kaplan–Meier analysis was used to investigate mortality and occurrence of VE, comparing groups with and without aPL in early disease.

**Results:**

Of 501 patients, 190 (38%) had at least one of these aPL, of whom 112 had anti-DI alone. Of 276 patients with complete vascular history, 83 had experienced VE. The 39 patients who were double or triple-ELISA-positive for any combination of the three aPL were more likely to have or develop lupus anticoagulant (*P*<0.0001) than those who were single-ELISA-positive or negative. In Kaplan–Meier analysis, they showed a trend towards developing more VE (*P* = 0.06).

**Conclusion:**

IgG anti-DI antibodies were present in early serum samples from 29% of patients and were more common than IgG aCL or anti-β2GPI. There was some evidence suggesting that double or triple-ELISA-positivity for these antibodies identified a group with worse outcomes.

**Rheumatology key messages**

- IgG anti-phospholipid antibodies were present in 38% of early-disease samples from patients with SLE.
- IgG antibodies to domain I (DI) of beta-2-GPI were the commonest specificity detected.
- Positivity for >1 of IgG anti-cardiolipin, anti-beta-2-GPI and/or anti-DI was associated with worse outcomes.

## Introduction

aPL were first described in the context of SLE and multiple reports over the last 30 years have shown that they are present in the blood of 20–30% of patients with SLE [1]. Only a minority of these patients, however, develop vascular thrombosis and/or pregnancy morbidity, which are the cardinal features of the APS [2]. There are three tests for aPL used commonly in clinical practice; the aCL ELISA, the anti-beta-2-glycoprotein I (anti- β 2GPI) ELISA and the LA test [2]. In many lupus clinics, these tests are requested routinely for screening in all newly diagnosed patients with SLE. It is not clear, however, what to do in the case of patients who have positive aPL results at diagnosis, whereas they have not yet suffered thrombosis or pregnancy loss. It is recognized that features such as high-titre positivity for IgG aCL and/or anti-β2GPI, and triple positivity for LA, aCL and anti-β2GPI [3, 4] are all factors that increase risk of thrombosis [5], but not to the extent that routine anticoagulation for those with these serological profiles is recommended as primary prophylaxis to prevent thrombosis.

β2GPI consists of five domains of which the N-terminal domain (Domain I, DI) is believed to contain the major immunological epitope [6, 7]. Thus, assays for anti-DI antibodies have been proposed as an alternative test that could have greater predictive power for occurrence of thrombosis and pregnancy morbidity [7–10]. However, there is little evidence concerning the prevalence of anti-DI antibodies early in the disease course of patients with SLE and whether presence of these antibodies with or without aCL and anti-β2GPI positivity delineates patient groups with higher risk of mortality or vascular events (VE) over many years of follow-up. The aim of this study was to investigate these unanswered questions.

We therefore extracted the first available stored serum sample for 501 patients with SLE, tested these samples for IgG aCL, IgG anti-β2GPI and IgG anti-DI, and compared these serological profiles to clinical outcomes of death or VE during the subsequent follow-up period.

## Methods

At University College London Hospital (UCLH), the specialist SLE clinic has been running since 1979 and has followed over 600 patients. Stored serum samples from these patients have been kept at −80°C. All patients entered into the lupus cohort at UCLH fulfil the American College of Rheumatology criteria for SLE [11]. The earliest available serum samples from 501 patients were retrieved from storage and used in the ELISAs. For over 90% of patients, this earliest sample was obtained within two years of diagnosis.

Clinical information on VE and LA positivity was obtained from patient records and from interviews with patients (for those still alive and under follow-up in the clinic) with informed consent and with ethics approval from the London Hampstead Research Ethics Committee (Reference Number 12/LO/0373).

VE were defined as including deep venous thrombosis, pulmonary embolism, catastrophic APS, myocardial infarction, stroke and coronary heart disease (the last two required proof by appropriate imaging).

### LA assay

LA is assessed at UCLH in accordance with national and international guidelines [12, 13]. Samples are processed within four h of collection and platelet-poor plasma is prepared from blood withdrawn by venepuncture in 0.109 M sodium citrate 9 : 1, then double-centrifuged at 1500 g for 15 min and stored at −80°C immediately after preparation. LA activity is confirmed in non-anticoagulated samples by the DRVVT, using Siemens Healthcare (Marburg, Germany) LA1 (screening) and LA2 (confirmation) reagents (Siemens Healthcare (Marburg, Germany). For patients receiving warfarin, the DRVVT includes testing with screen and confirm reagents on equal volume mixtures of patient/normal plasma (which, if positive, confirms the presence of an inhibitor and phospholipid dependence) and a Taipan venom time (TVT)/Ecarin clotting time (Diagnostic Reagents Ltd, Thame, UK) ratio is also performed. The normalized ratio cut-off value for the DRVVT is 1.20 and for the Taipan venom time/Ecarin clotting time 1.12.

### ELISA

The methods for the IgG aCL, IgG anti-β2GPI and IgG anti-DI ELISAs have been detailed fully in previous papers [9, 14, 15]. For this study, we had established positive/negative cut-offs in all three of these ELISAs as the 99th percentile of 40 healthy controls. These levels are 21 GPLU for aCL, 22 GBU for anti-β2GPI and 22 GDIU for anti-DI.

In this paper we use the term single-ELISA-positive for any patient positive for either IgG aCL or IgG anti-β2GPI or IgG anti-DI, double-ELISA-positive for any patient positive in any two of these ELISAs and triple-ELISA-positive for any patient positive in all three ELISAs. These terms are used to make a clear distinction with the terms double and triple-positive that are generally used in APS to refer to aCL, anti-β2GPI and LA [3, 4].

### Statistical analysis

For the Kaplan–Meier analysis of mortality, patients were divided on the basis of serology of the initial sample into aPL-single-ELISA-positive, aPL double/triple-ELISA positive and aPL-negative. Patients were censored at the time of loss to follow-up or at the end of the study period. For the Kaplan–Meier analysis of VE, patients were censored at the time of death, loss to follow-up or the end of the study period.

## Results

For the 501 patients with SLE included in the study, mean age at the time of sample was 30 years (s.d. 12.2) and mean follow-up was 12.1 years, with a maximum of 36 years. There were 457 (91%) women and 307 (67%) Caucasians. For the 40 healthy controls, mean age was 31.3 years (s.d. 7.1), 26 (65%) were women and 33 (83%) Caucasian. Table 1 shows demographic, clinical and serological features of these patients.

**Table 1:** Demographic, clinical and serological features of patients in this study

Age, mean (s.d.), years	30.0 (12.2)	Range: 1–77
Female gender	457	91.2%
Ethnicity		
Caucasian	309	61.7%
Asian	84	16.8%
African/Caribbean	95	19.0%
Other	13	2.6%
Skin disease		
Rash	342	68.3%
Photosensitivity	217	43.3%
Alopecia	132	26.4%
Mouth ulcers	133	26.6%
Joint disease	475	94.8%
Kidney disease	181	36.1%
Serositis	209	41.7%
Central nervous system disease	99	19.8%
Positive anti-dsDNA (ever)	334	66.7%
Low complement (ever)	250	49.9%
Anti-extractable nuclear antigens (ever positive)		
Anti-Sm	76	15.2%
Anti-Ro	197	39.3%
Anti-La	71	14.2%
Anti-RNP	148	29.5%
Rheumatoid factor	124	24.8%

### Prevalence of IgG aCL, IgG anti-β2GPI and IgG anti-DI positivity at baseline

**Fig. 1:**
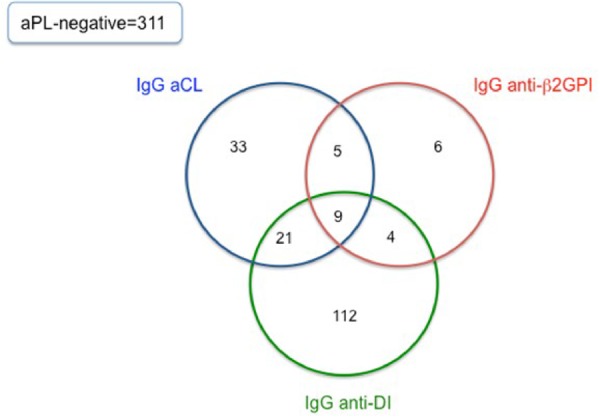
Prevalence of IgG anti-β2GPI, aCL and anti-DI in 501 samples from patients with SLE The Venn diagram displays the number of single-ELISA, double-ELISA and triple-ELISA positive IgG anti-β2GPI, aCL and anti-DI in 190 patients with SLE. The number of aPL-negative samples is also shown. anti-β2GPI: anti-beta-2-glycoprotein I; DI: domain I.

Of the 501 samples from early disease, 190 (38%) were positive for one or more of IgG aCL, IgG anti-β2GPI and IgG anti-DI. A total of 39 patients were double-ELISA-positive for any of the three aPL, or triple-ELISA positive. The Venn diagram in Fig. 1 shows how the populations seropositive for these antibodies overlapped. IgG anti-DI was the most common antibody found (146/501 patients, 29%) whereas IgG anti-β2GPI was the least common (24/501, 5%). In fact, 112 patients (22%) were single-positive for anti-DI alone. Correlation was stronger between aCL and anti-β2GPI (correlation coefficient = 0.60) than between anti-DI and anti-β2GPI (0.19) or anti-DI and aCL (0.12). The mean IgG anti-DI level was higher in those who were positive for both IgG anti-DI and IgG anti-β2GPI than in those who were positive for IgG anti-DI alone (49.5 *vs* 24.2 GDIU, *P* = 0.04).

The Sydney criteria [2] use definitions of aCL positivity higher than ours (>40 GPLU).

Of 68 patients whose samples were positive for IgG aCL, 32 had values > 40 GPLU. Of 24 positive for IgG anti-β2GPI, eight had values > 40GBU. Of 146 IgG anti-DI-positive patients, 46 had values > 40 GDIU.

### Presence of IgG aPL in early disease is strongly associated with development of LA

In 216 cases, we had data on LA positivity as well as early aPL serology. Of those, 177 (82%) were LA-negative and 39 (18%) were LA-positive at some point either at diagnosis or during the follow-up period. Positivity for any of the three aPL in the earliest sample was significantly more common in LA-positive than LA-negative patients [25/39 (64%) *vs* 55/177 (31%), *P* = 0.0002]. Furthermore, 9/39 (23%) of LA-positive patients were double-ELISA or triple-ELISA-positive for IgG aCL, anti-β2GPI and/or anti-DI in the earliest sample compared with 5/177 (3%) of LA negative patients (*P* <0.0001).

### Survival analysis of the effect of different antibody profiles at baseline

#### Mortality

Of the 501 patients for whom serological data were available, 67 (13%) had died, of whom 34 were aPL-positive. Thus 34/190 (17.8%) aPL-positive *vs* 33/311 (10.6%) aPL-negative patients had died (*P* = 0.022 Fisher’s exact test). Nine patients who died were double-ELISA or triple-ELISA-positive.

Causes of death in the aPL-positive group were; cancer 12 (35.3%), infection 7 (20.6%), vascular 7 (20.6%), other 8 (23.5%). Causes of death in the aPL-negative group were; cancer 6 (18.2%), infection 9 (27.3%), vascular 5 (15.2%), other 13 (39.4%).

**Fig. 2:**
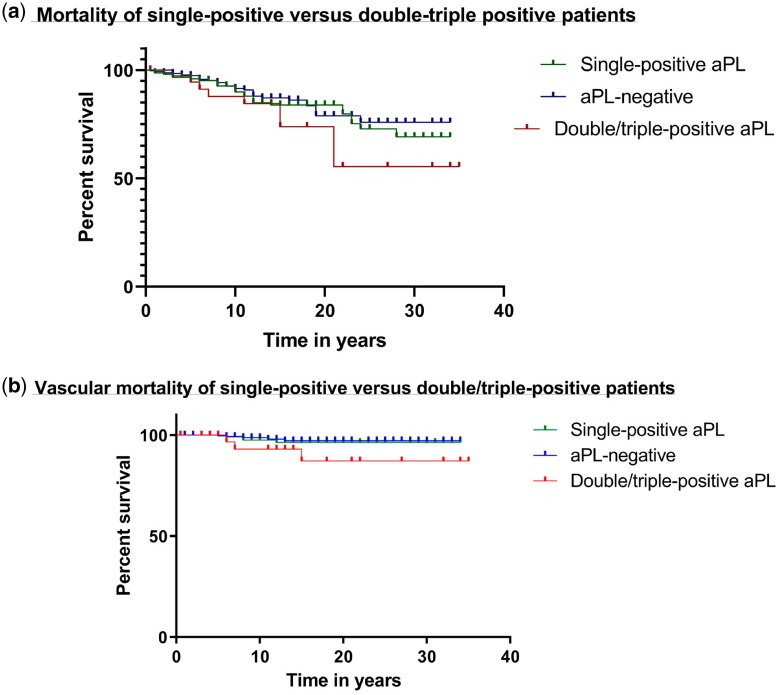
Mortality and vascular mortality over time in patients stratified by serology for aPL This figure shows survival in all 501 patients in the study over time. Fig. 2a includes all deaths and Fig. 2b includes only vascular deaths. The x-axis shows time in years.

Fig. 2 shows survival curves for patients divided into three groups – aPL-negative, single-ELISA-positive and double/triple-ELISA-positive based on the results in the initial sample. Fig. 2a includes all deaths whereas Fig. 2b includes only vascular deaths on the basis that these are more likely to be linked to aPL. There was no statistically significant difference between the curves in either figure, though at time points after 15 years the double/triple-ELISA curve falls below the others in both figures.

The numbers remaining under follow-up were 438 (168 in the original aPL-positive group) at 5 years, 284 (127) at 10 years, 179 (86) at 15 years, 101 (57) at 20 years, 48 (27) at 25 years and 25 (16) at 30 years.

#### Vascular events

We had both aPL serology in the earliest sample and complete history of VE for a sub-group of 276 patients (25 men). Of these patients, 112 (41%) were positive for at least one of IgG aCL, IgG anti-β2GPI and IgG anti-DI in the original sample.

Eighty-three of these 276 patients had suffered a VE, including 43/112 (38%) in the aPL-positive group, 12/21 (57%) in the double/triple-ELISA-positive group and 40/164 (24%) in the aPL-negative group. The types of VE in each group are shown in Table 2.

**Table 2:** Types of VE in different groups of patients

Type of VE	aPL-negative (*n* = 164) *n* (%)	Positive for any aPL (*n* = 112) *n* (%)	Double or triple ELISA positive (*n* = 21) *n* (%)
Any VE	40 (24%)	43 (38%)	12 (57%)
Venous thrombosis alone	19 (12%)	14 (12.5%)	6 (29%)
Stroke alone	12 (7%)	12 (11%)	3 (14%)
IHD alone	6 (4%)	10 (9%)	0
Venous thrombosis and stroke	0	2 (2%)	2 (9.5%)
Venous thrombosis and IHD	0	3 (3%)	1 (5%)
Stroke and IHD	3 (2%)	2 (2%)	0

IHD: ischaemic heart disease; VE: vascular events.

Twenty-five (10 aPL-positive, four double/triple-ELISA-positive) patients suffered the VE before the date of the earliest sample and were censored at a value of 0.1 years in the Kaplan–Meier analysis. The numbers remaining under follow-up were 210 (92 in the original aPL-positive group) at 5 years, 122 (59) at 10 years, 73 (42) at 15 years, 35 (27) at 20 years, 15 (12) at 25 years and 8 (7) at 30 years. Of the 33 aPL-positive patients who suffered VE during the follow-up period, only three were on anticoagulants at the time of their first VE, 23 were definitely not anticoagulated and for the other seven, this information was unavailable.

**Fig. 3:**
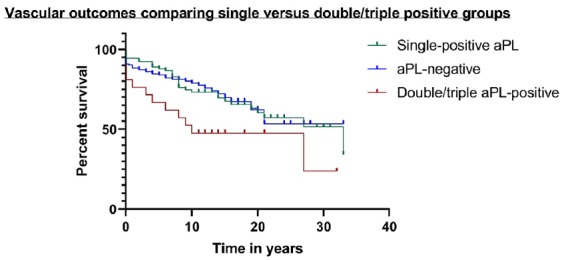
Incidence of vascular events over time in patients stratified by serology for aPL This Kaplan–Meier curve shows vascular event-free survival analysis of 276 patients for whom both full vascular history and aPL results in samples from early disease were available. Patients were stratified into three groups: aPL-negative; single-ELISA-positive; and double/triple-ELISA-positive. The x-axis shows time in years.

Fig. 3 shows a Kaplan–Meier graph demonstrating VE outcomes in different groups of patients stratified by the serology in the earliest sample. The curve comparing double/triple-ELISA positive patients to the other groups shows a clear divergence of the curves, with the double/triple-ELISA group having the worst outcomes, though the *P*-value just failed to reach significance (*P* = 0.06). We also compared aPL-positive (any) to aPL-negative, anti-DI-positive to anti-DI-negative and high anti-DI positive (>40 GDIU) to low-anti-DI positive and anti-DI negative and no differences between groups were seen.

## Discussion

The strengths of this analysis are the large number of individual patients studied and the long-term follow-up of mortality and VE, as shown in the Kaplan–Meier analysis. The major findings were the high proportion of patients positive for IgG anti-DI in early disease and a trend towards increased risk of developing VE in patients who were either double or triple-ELISA-positive in early disease.

The role of testing for anti-DI in diagnosis of APS has been tested in a number of studies. In a meta-analysis of 11 studies including 1218 patients with APS, 318 patients with SLE, 49 asymptomatic aPL-positive individuals and 1859 healthy controls, Radin *et al.* [16] reported that 45.4% of patients with APS were positive for anti-DI and that four of the five studies that looked at association between anti-DI-positivity and risk of thrombosis found such an association, with odds ratios ranging between 2.5 and 4. Other studies, however, have noted inconsistency of results obtained using different techniques for measuring anti-DI [17] and concluded that the anti-DI assay does not add to the predictive value for thrombosis of the combination of three assays used in current clinical practice [17–20].

A large number of patients in our study tested positive for IgG anti-DI in samples taken very early in disease. There are two surprising things about this result. Firstly, several other papers have shown that IgG anti-DI positivity is less common than IgG aCL or anti-β2GPI positivity in patients with SLE [9, 18, 19, 21], leading to the argument that anti-DI is more specific for APS but less sensitive [17–20]. A different result was obtained by Wahezi *et al.* [22] in a population of 183 children with SLE, of whom 25.1% were IgG anti-DI positive, though that paper did not report on presence of anti-β2GPI antibodies and only a minority of patients were tested for IgG aCL.

Secondly, 112 patients in our study were positive for IgG anti-DI but negative for aCL and anti-β2GPI. There was very low correlation between IgG anti-β2GPI and IgG anti-DI titres. This is very different to results obtained in previous studies in which these antibody specificities are strongly correlated [18, 19] as would be expected from the fact that DI is part of β2GPI. For example, De Craemer *et al.* [19] tested 426 subjects and found that of 60 who were IgG anti-β2GPI-positive, 55 were also IgG anti-DI-positive. Iwaniec *et al.* [18] tested 103 patients with APS, 99 with SLE and 102 healthy control subjects and found that of 109 who were IgG anti-β2GPI-positive, 78 were also IgG anti-DI-positive. Only one patient was IgG anti-DI-positive but IgG anti- β 2GPI-negative.

One possible explanation for this apparently high level of isolated anti-DI positivity in our study is that it arises for methodological reasons. As described by Yin *et al.* [17] in a recent review, both IgG anti-β2GPI and IgG anti-DI assays give variable results depending on factors such as source of antigen, coating density of antigen and exposure of the immunogenic G40-R43 epitope on DI. Thus, it is possible that our anti-β2GPI ELISA gives falsely low results because that epitope is not fully exposed. Alternatively, if the positive cut-off for IgG anti-DI in this study (established in a relatively small number of healthy control subjects) is too low, there might be false-positive readings. It is impossible to exclude either of these possibilities completely, although in our paper comparing results from 111 patients with APS, 119 patients with SLE and 200 healthy controls, the specificity of the IgG anti-DI ELISA for APS was 96% [9].

The majority (100/146) of the IgG anti-DI-positive patients have low positive titres (between 21 and 40 GDIU) suggesting antibodies of low affinity. Arbuckle *et al.* [23] studied samples from 130 American military personnel that had been stored many years before they were diagnosed with SLE and found that autoantibodies were often present before clinical manifestations of the disease had developed. aPL were among the earliest specificities detected. It is possible that early in the disease course of our 501 patients, low affinity anti-DI antibodies are present. These antibodies could potentially bind to DI coated on an ELISA plate much better than whole β2GPI, where the key epitope on DI is not accessible in all molecules. As the disease progresses, some patients might lose these low affinity antibodies altogether, while others develop higher affinity anti-DI. However, because we do not have IgG anti-DI data from later time points, this idea is speculative.

Although this study shows that 38% (190/501) of patients had at least one of these three aPL very early in disease, the analysis does not suggest that aPL-positivity *per se* predicts clinical outcomes. Kaplan–Meier analysis of mortality or vascular mortality did not show significant differences between aPL-positive and aPL-negative patients and causes of death in aPL-positive patients were not skewed towards VE.

The one subgroup of patients identified as having a potentially worse long-term outlook in this study were those who tested positive for either two or three of IgG aCL, anti-β2GPI and anti-DI early in disease. There were 39 such patients, but if anti-DI had not been tested, only 14 of these would have been identified (i.e. aCL plus anti-β2GPI positive). The Kaplan–Meier curves suggest that this double/triple-ELISA positive group do worse in terms of vascular outcomes, but this result cannot be over-interpreted because even in this large group of patients, the results did not reach statistical significance. There was, however, a strong association between double/triple-ELISA positivity and LA. Pengo *et al.* [24] noted that while there are some patients who test LA-positive but anti-β2GPI negative (due to presence of IgM anti-phosphatidylserine/prothrombin antibodies), those positive in both assays have a much higher risk of thrombosis.

Limitations include the fact that this study is cross-sectional, with ELISA data for a single time-point for each patient, and that VE were recorded retrospectively from patients’ charts and interviews. It is not possible to test LA in stored serum samples because of the method used in the LA assay, which requires the blood to clot. Only IgG isotype antibodies were tested. In future studies it would be of interest to obtain samples taken from these same serological groups of patients at later dates to assess the extent to which single, double and triple-ELISA positivity remain stable over time.

In summary, IgG anti-DI antibodies were found in early serum samples from 29% of 501 patients with SLE and were more commonly detected than IgG anti-β2GPI and aCL. There was no strong evidence that testing for IgG anti-DI in early disease would alter management of patients with SLE.
